# Profiling Animal Toxicants by Automatically Mining Public Bioassay Data: A Big Data Approach for Computational Toxicology

**DOI:** 10.1371/journal.pone.0099863

**Published:** 2014-06-20

**Authors:** Jun Zhang, Jui-Hua Hsieh, Hao Zhu

**Affiliations:** 1 Department of Chemistry, Rutgers University, Camden, New Jersey, United States of America; 2 The Rutgers Center for Computational and Integrative Biology, Camden, New Jersey, United States of America; 3 Biomolecular Screening Branch, Division of National Toxicology Program, National Institute of Environmental Health Sciences, Research Triangle Park, North Carolina, United States of America; University of Memphis, United States of America

## Abstract

*In vitro* bioassays have been developed and are currently being evaluated as potential alternatives to traditional animal toxicity models. Already, the progress of high throughput screening techniques has resulted in an enormous amount of publicly available bioassay data having been generated for a large collection of compounds. When a compound is tested using a collection of various bioassays, all the testing results can be considered as providing a unique bio-profile for this compound, which records the responses induced when the compound interacts with different cellular systems or biological targets. Profiling compounds of environmental or pharmaceutical interest using useful toxicity bioassay data is a promising method to study complex animal toxicity. In this study, we developed an automatic virtual profiling tool to evaluate potential animal toxicants. First, we automatically acquired all PubChem bioassay data for a set of 4,841 compounds with publicly available rat acute toxicity results. Next, we developed a scoring system to evaluate the relevance between these extracted bioassays and animal acute toxicity. Finally, the top ranked bioassays were selected to profile the compounds of interest. The resulting response profiles proved to be useful to prioritize untested compounds for their animal toxicity potentials and form a potential *in vitro* toxicity testing panel. The protocol developed in this study could be combined with structure-activity approaches and used to explore additional publicly available bioassay datasets for modeling a broader range of animal toxicities.

## Introduction

The evaluation of chemical toxicity by traditional animal testing protocols has proven prohibitively costly and time consuming for screening large numbers of chemicals. A wide range of *in vitro* bioassays are being developed and used as potential alternatives to traditional animal models. In the past decade, the progress of high throughput screening (HTS) techniques has resulted in the generation of enormous amounts of bioassay data for large collections of compounds. For example, PubChem, which is the largest public data source [1], contained nearly 47 million deposited compounds at the end of 2012. Among them, more than 1,620,000 compounds have been tested by over 500,000 bioassays and thus there are more than 130 million bioactivity outcomes (either actives, inactives or inconclusives) from these bioassays on PubChem [2]. The generation of bioassay data is at an unprecedented scale and the so called “big data” approaches are required to analyze all the available data [3] [4].

Many compounds (e.g., pesticides) of environmental or pharmaceutical interest have been tested against multiple bioassays for various purposes. For example: dichloro-diphenyl-trichloroethane (DDT, CID 3306) has been tested against 383 PubChem assays as of August, 2013, and it shows active responses in 28 assays. A collection of data from these assays, especially the assays with active responses, would provide useful response information to study the potential toxicity responses of DDT *in vivo*. If a compound is tested against a panel of bioassays, the test results can be considered as a response profile for this compound. Likewise, if a collection of chemicals is tested against the same panel of bioassays, the response profiles of the chemical collection constitute a set of common biological descriptors that reflect the results of compounds interacting with different biological receptors. Specifically, if the receptors of two or more bioassays are potentially relevant to toxicity (i.e., two receptors belong to a toxicity pathway), the response profiles contain potential perturbation information of the tested compounds with respect to both assays. This information is helpful for studying the systemic chemical perturbation to the whole biological system. In this way, experimentally profiling compounds of environmental interest based on relevant bioassay data has been used to study complex animal toxicity [5].

Several recent efforts have attempted to use *in vitro* HTS profiling to experimentally screen chemicals for potential toxicity. For example, in 2006 the U.S. EPA initiated the “ToxCast” program with the goal of developing methods for utilizing diverse *in vitro* HTS technologies to quickly screen for potential toxicity and to prioritize candidates for future animal testing [6]. In the first phase of this program, 309 well-characterized chemicals (primarily pesticides) were screened against around 600 bioassays using multiple HTS technologies. The ToxCast chemical library has more recently expanded to over 1,800 compounds, spanning a wide range of chemical use categories and structural classes, with full data sets yet to be released [7] [8]. In a related, multi-federal-Agency effort, the Tox21 project is screening a chemical library consisting of over 8,000 unique chemicals in panels of quantitative HTS (qHTS) assays being developed at the NIH Chemical Genomics Center (NCGC) [9]. In the early phases of this program, the National Toxicology Program (NTP) tested an initial set of 1,400 compounds across several cell-based assays having potential relevance to toxicity. These qHTS data, currently spanning several hundred bioassays, are publicly available in PubChem in collaboration with the NCGC.

Acute toxicity is defined as the adverse effects occurring following a short period of exposure or a short time of administration of chemical substances [10]. In 2009, a collaborative toxicological study aimed to develop and validate the use of *in vitro* methods for the prediction of human acute toxicity. This study, undertaken by several research organizations in Europe [11], established that a set of 100 bioassays correlated with acute toxicity for a set of 97 compounds. This latter study focused on a relatively small set of compounds and a limited number of bioassays were used.

Several virtual screening studies aimed to extract useful bioassay information from public data sources for specific compounds were also reported. For example, Rohrer and Baumann developed a workflow to construct a maximum unbiased validation (MUV) data set from multiple bioassay collections of PubChem for virtual screening purposes [12]. Butkiewicz et al. selected nine datasets from the confirmatory HTS data of PubChem to benchmark computer-aided drug discovery studies [13]. Schierz studied the effect of false positive problems of the PubChem bioassay on virtual screening [14]. Moreover, the PubChem bioassay data have been used in various modeling studies: a decision tree approach using the HTS data [15]; a Bayesian approach using the HTS data [16]; a support vector machine (SVM) approach for inhibitor or ligand classifications [17]; and a GPU accelerated SVM approach [18]. In 2010, Xie reviewed the previous applications of bioassay data in PubChem and he also addressed two major challenges in the application of PubChem bioassay data: a biased active/inactive ratio and experimental errors shown as false positives or negatives [19]. Overall, previous studies using public bioassay data (i.e., PubChem) required extensive manual data curation.

Due to the nature of manual selection of bioassays from public resources, the total amount of data that could be analyzed is limited in the above-mentioned studies. In these studies, the correlation between a small set of bioassays with targeted complex bio-activities (e.g. animal toxicity) is somewhat arbitrary because of the manual selection. Furthermore, since there is no standard criterion for representation and annotation of public bioassay data, the quality and format of the available data may vary from different sources and greatly affects the potential usefulness of these bioassay results. Thus, an automatic data mining method is necessary to extract, integrate, and evaluate bioassays from public data sources. Automatically selected bioassays could be used in future studies to profile compounds of particular biological interest.

In this study, we developed a new data mining method to extract information rich bioassays from PubChem that could be used to generate response profiles for the evaluation of animal acute toxicity of compounds. The protocol developed in this study is entirely general and could be applied to processing other big data type public sources (such as being generated in ToxCast and Tox21), as well as to the profiling of other complex bioactivities (i.e., other animal toxicities).

## Materials and Methods

### Publicly Available Bioassays

Public bioassay data was obtained from the PubChem repository developed by the National Center for Biotechnology Information (NCBI). As of February, 2014, there were 739,668 bioassays recorded in this repository. These assays were either collected from other bioassay databases, such as ChEMBL [20], or deposited by various laboratories such as the screening centers of the Molecular Libraries Program (MLP) of the National Institutes of Health (NIH) (http://www.mli.nih.gov/mli/). For example, the NCI60 bioassays, consisting of 60 human tumor cell lines, were developed by the Developmental Therapeutics Program of the National Cancer Institute (DTP/NCI) and used as an anticancer drug screen panel [21]. Each bioassay has a unique PubChem assay identifier (AID). The number of compounds tested in every bioassay ranges from 1 (e.g., AID 569473) to more than 400,000 (e.g., AID 602332). Although many bioassay data were originally deposited as continuous data, we used the PubChem classification results (active, inactive, and unspecified/inconclusive) in our study to simplify the data mining procedure.

### Profiling Compounds with PubChem Bioassays

#### 1 Identifying target compounds using the PubChem compound accession identifier

Normally compounds collected from various sources have their own identifiers, such as CAS Registry numbers (http://www.cas.org/content/chemical-substances/faqs, Accessed June 1, 2013), and ZINC ID (http://zinc.docking.org/, Accessed June 1, 2013). All these identifiers are used by various chemical sources, which normally contain millions of compounds. For PubChem, each compound has a unique compound accession identifier (CID) linked to a unique chemical structure. When a compound is listed on PubChem, there is usually a formal chemical name, synonyms, which includes trivial names, brand names, IUPAC-like name, chemical formula, and basic physical properties (e.g., molecular weight). Among all this information, only CID can be used to identify a unique compound by its chemical structure. For this reason, all the compounds in our dataset were initially identified by their CIDs. This procedure was finished by automatically transferring other existing identifiers (e.g., CAS) in our dataset into CIDs or automatically searching for a corresponding CID by the chemical structures.

#### 2 Generating the initial response profiles

The PubChem compounds can be virtually linked to their bioassay records through Entrez Utilities (http://www.ncbi.nlm.nih.gov/books/NBK25500/, Accessed June 1, 2013). Entrez Utilities were developed by the NCBI to let users query various databases through a web browser. We developed programs to perform on-line data extraction from PubChem by embedding the keywords of Entrez Utilities into Perl scripts, which are provided in **Protocol S1**.

The number of compounds that show active results in a HTS bioassay is often much less than the number of inactives. Thus, a bioassay with very few active responses, which indicates little response information recorded by this assay in our target compounds, was excluded first. We used an arbitrary cutoff, which ensures at least 6 actives in each selected bioassay among the target compounds, to remove those somewhat “insignificant” bioassays that provide little information.

After removing insignificant bioassays, we can build an *m×n* dimension matrix *M*, as the initial response profiles for our target compounds. This matrix consists of *m* bioassays as columns and *n* compounds as rows. The cell *M_ij_* of *M* has a value of either 1 (indicates compound *j* is active in assay *i*), −1 (indicates compound *j* is inactive in assay *i*), or 0 (indicates compound *j* is inconclusive or untested in assay *i*). An assay in this matrix could also be considered as a vector *A_i_* (*i* = 1–*n*) with *n* dimensions.

#### 3 Selecting the most useful bioassays for the targeted compounds’ animal toxicity

It is clear that not all the initially selected PubChem bioassays are useful to the type of animal toxicity that we want to study (i.e., animal acute toxicity). To assess informational or statistical relevance, we evaluated all the bioassays in the initial response profiles based on their correlation to our target animal toxicity. Thus, the animal toxicity results could also be constructed as an extra vector *T* with the same dimensions of assay vector *A_i_*. We used two parameters to select the most relevant bioassays. Firstly, the correct classification rate (CCR) between animal toxicity vector *T* and *A_i_* (*i* = 1–*n*) is calculated as:
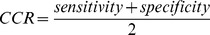
(1)

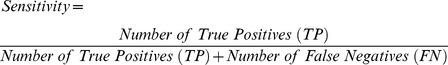
(2)

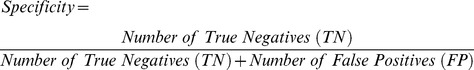
(3)


A True Positive (TP) is defined when a compound is active in both *T* and *A_i_*. A True Negative (TN) is defined when a compound is inactive in both *T* and *A_i_*. A False Positive (FP) is defined when a compound is active in *A_i_* but inactive in *T*. A False Negative (FN) is defined when a compound is inactive in *A_i_* but active in *T*. An assay with high CCR value indicates that the correlation between this assay and the target animal toxicity (animal acute toxicity in this study) is high.

Secondly, we defined a parameter *L(A_i_)* to calculate the likelihood of correlation between the activities of bioassay and animal toxicity for the targeted compounds as follows:
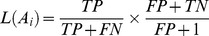
(4)


The higher *L(A_i_)* value for a bioassay indicates this bioassay has higher correlation with animal toxicity. The parameter *L(A_i_)* gives higher weights to the True Positive Rate (TPR) rather than the True Negative Rate. Thus, if the number of FP compounds increases, the *L(A_i_)* value will significantly decrease.

#### 4 Evaluating the animal acute toxicity potentials of chemicals using selected bioassay data

The activity of a compound in PubChem bioassays is categorized by active (represented as 1 in this study), inactive (represented as −1) or inconclusive/untested (represented as 0). If one compound was tested using multiple assays, the consensus activity was calculated by evaluating the individual response of this compound in all tested bioassays. Considering that any given compound usually was tested in assays from different sources and protocols, we defined a new parameter, called *S* score, to rank the toxicity potential of compounds from multiple bioassays as follows:



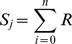


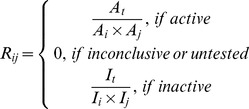
(5)


Among the above equations, the *S* score for compound *j* is defined as the sum of the normalized response parameter *R* for each individual bioassay. When the response of assay *i* is active, *R* is normalized by considering the total number of active responses (*A_t_*) in *M*, the total number of active responses in this bioassay (*A_i_*), and the total number of active responses in this specific compound in all the bioassays (*A_j_*). Similarly, when the response of assay *i* is inactive, *R* is normalized by considering the total number of inactive responses (*I_t_*) in *M*, the total number of inactive responses in this bioassay (*I_i_*), and the total number of inactive responses in this specific compound in all the bioassays (*I_j_*). The *R* is zero when there is no test data or the response is “inconclusive”.

**Figure 1:**
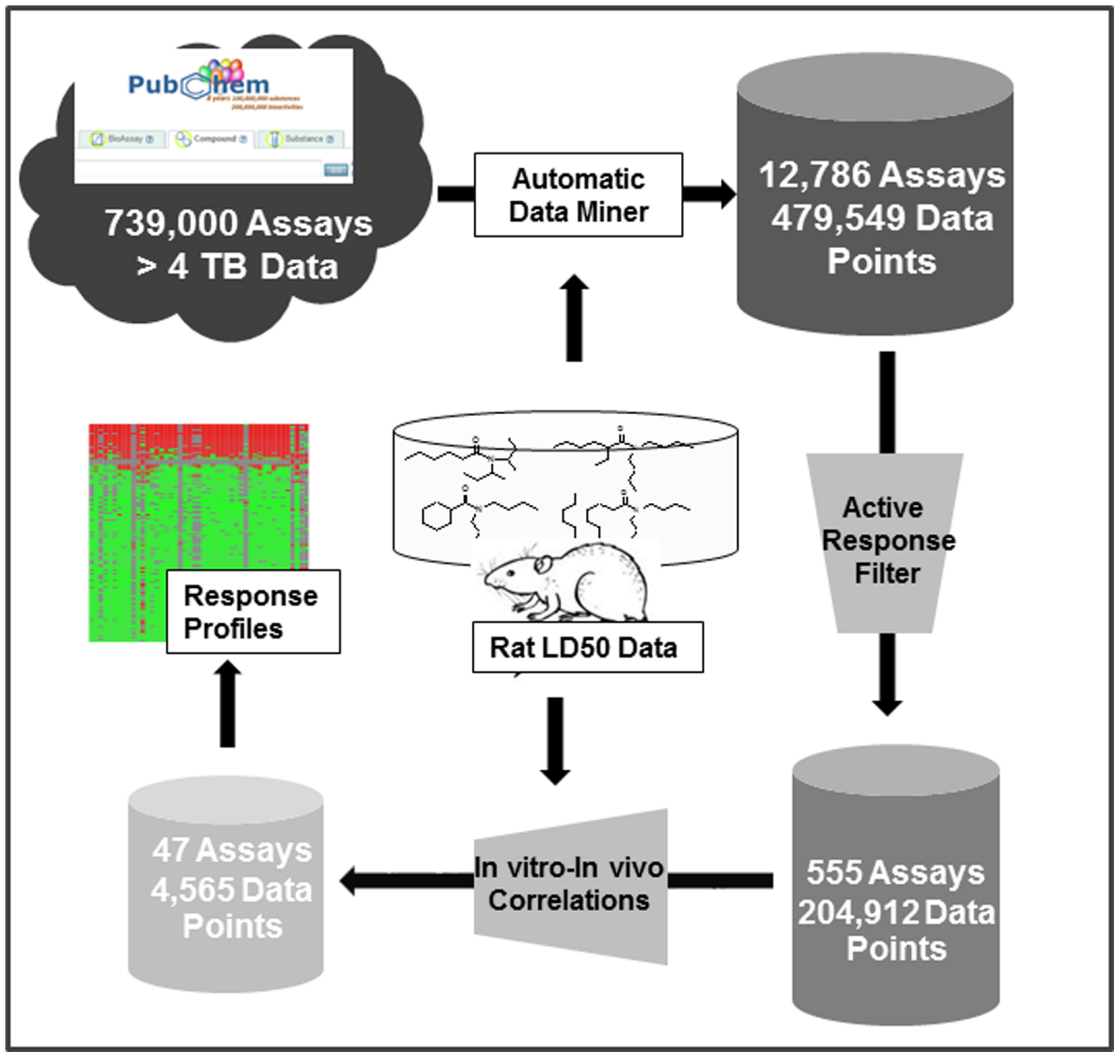
The automatic response profiling based on *in vitro-in vivo* relationship.

In summary, the whole profiling procedure, which lies at the core of this paper (**Figure 1**), is to automatically mine the public big data resources for the compounds of specific interest. The response profiles, obtained from the *in vitro-in vivo* relationship analysis, could be viewed as the potential toxicity mechanisms of toxicants and be used to prioritize candidates for future animal testing.

### Rat and Mouse LD_50_ Dataset

The rat acute toxicity data collection is described in our previous publication [22]. It is comprised of 7,385 unique organic compounds with rat *LD_50_* values expressed as a negative logarithm in units of moles per kilogram. Since we only consider the classifications of the bioassay data, the acute toxicity results of these compounds were also classified as “toxic” when *–log_10_LD_50_*(mol/kg) > 3.00, and “nontoxic” when *–log_10_LD_50_*(mol/kg) < 2.00. The compounds with *–log_10_LD_50_*(mol/kg) values between 2.00 and 3.00 are marginal. Among 7,385 compounds, there are 1,916 compounds deemed toxic, 2,544 marginal compounds, and 2,925 nontoxic compounds. This classification cut-off for animal acute toxicity was also used in previous studies based on acute toxicity guidelines [23]. Marginal compounds were removed initially, so there are totally 4,841 compounds being used in this study.

## Results and Discussions

### The Overview of PubChem Bioassay Data for the Target Compounds

The total number of PubChem bioassays initially used for profiling was over 739,000 and the size of data was over four terabytes (**Figure 1**). After we removed marginal toxicity compounds, there were a total of 4,841 unique organic compounds with their *LD_50_* data. Among these compounds, 3,840 compounds can be found in PubChem and have a unique structure-matched CID. Using these CIDs, we extracted 12,786 bioassays in which at least one of these compounds showed active responses. This effort resulted in 1,993 out of the 3,840 compounds with 479,549 data points. As mentioned above, we removed bioassay with less than 6 active compounds per assay. As the result, there were a total of 555 bioassays in the preliminary response profiles (204,912 data points) for 1,899 compounds (**Figure 1**).

**Figure 2:**
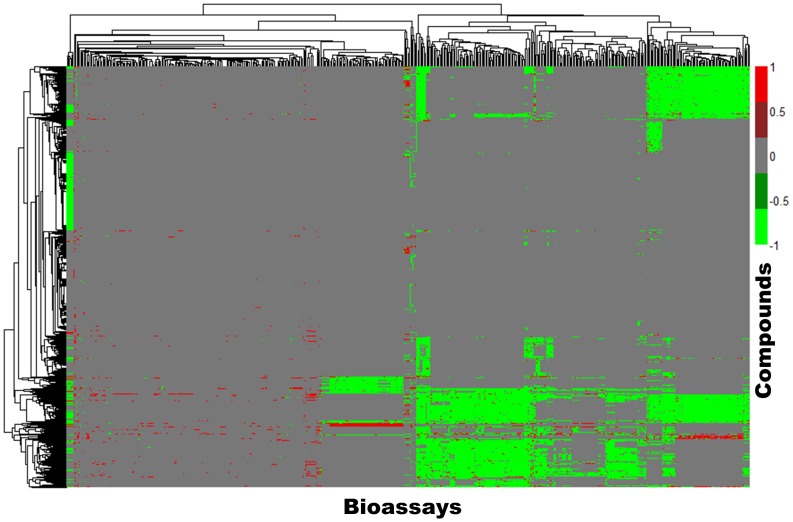
The heat map for the response profiles of 1,899 compounds against 555 bioassays. The red dot (Value = 1) indicates the compound has an active response in the corresponding bioassay. The light green dot (Value = −1) indicates the compound has an inactive response in the corresponding bioassay. The grey dot (Value = 0) indicates the compound is untested or has inconclusive results in the corresponding bioassay.

The response matrix (*M*) based on the initial response profiles of the 1,899 compounds can be viewed as a heatmap (**Figure 2**). Not surprisingly the majority of the responses in the initial response profiles are either null/inconclusive (represented as “0”) or inactive (represented as “−1”). The ratio of the active responses (represented as “1”) in this initial response heatmap is very low, which also reflects the nature of most HTS data. However, it is clear that the initial *M* only reflects responses of the target compounds in all tested PubChem bioassays. It is necessary to develop automatic tools to search for the most relevant bioassays to our target animal toxicity.

### Searching for Bioassays Useful to Animal Toxicity

**Figure 3:**
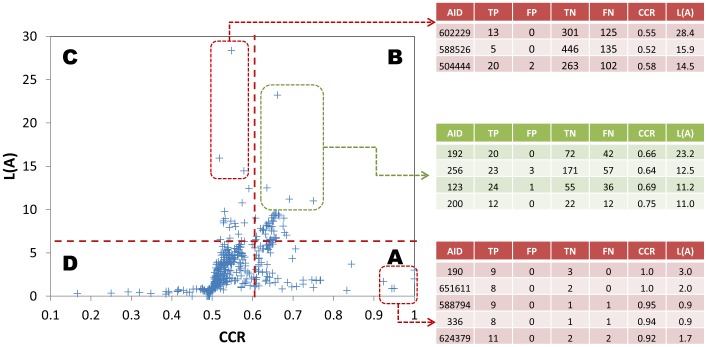
The distributions of 555 bioassays against their CCR values and the *L* parameters with animal acute toxicity. The graph could be divided into 4 areas by using two cutoff values (CCR = 0.6 and *L* = 7): 1) A indicates low *L* and high CCR; 2) B indicates high *L* and high CCR; 3) C indicates high *L* and low CCR; and 4) D indicates low *L* and low CCR. The tables on the right side displayed the counts of TP, FP, TN, FN along with CCR and *L* values for some bioassays from area A, B and C, respectively.

The relevance between animal acute toxicity and each of the 555 initial bioassays was calculated and ranked using both the CCR and *L* parameters described above. **Figure 3** shows the distribution of 555 bioassays against their CCR and *L* parameters. The table on the right side displays the values of TP, FP, TN, FN along with CCR, and *L* parameters for some representative bioassays (**Figure 3**). If only CCR is used as the ranking criteria, the top bioassays will have very limited TP and TN counts, as shown in the examples in area A of **Figure 3**. It is understandable that one individual bioassay is not able to fully replicate the complex toxicity mechanisms of animal toxicity and, hence, the results from a single bioassay are not able to correlate with the animal toxicity perfectly for all compounds. For this reason, only using the CCR as the parameter to rank bioassays will result in biased results (i.e. accidental selection of the bioassays with few TP and TN counts).

**Figure 4:**
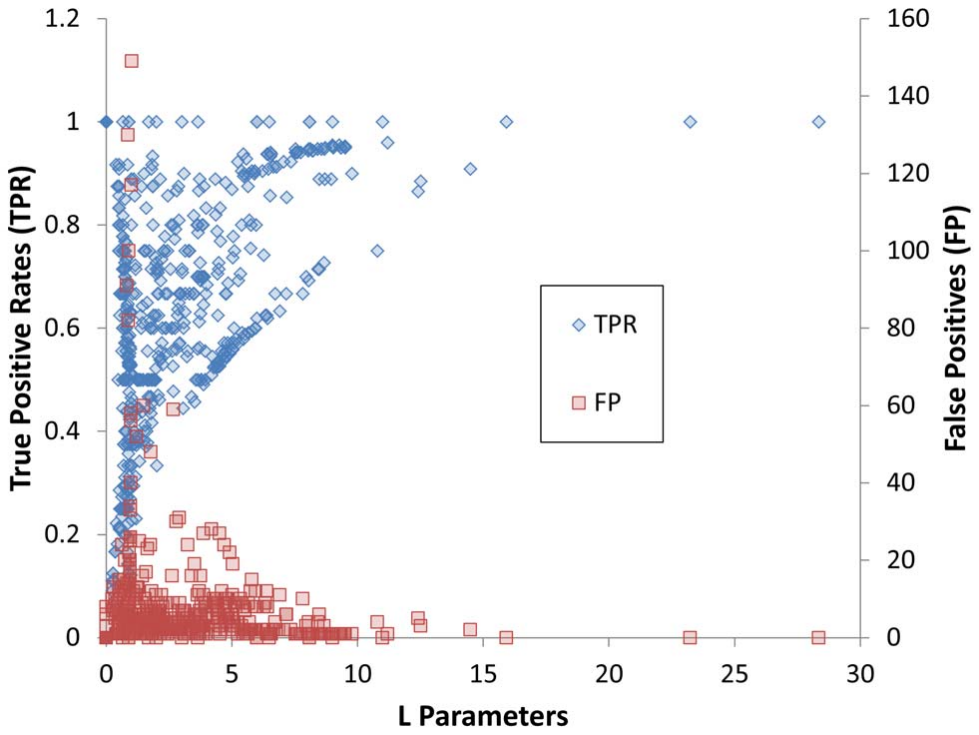
The relationships between *L* parameters and 1) the True Positive Rate (blue dots); and 2) the number of total False Positives (red dots) for 555 bioassays.

Considering the systemic effect of potential toxicants, we need novel methods to evaluate the correlations between bioassays and target animal toxicity. Our hypothesis based on systemic effects is that if the bioassay includes a receptor that contributes to a toxicity pathway and is relevant to the target animal toxicity, this bioassay should provide useful information relative to the target animal toxicity. However, if compounds show inactive results in a particular bioassay, it does not necessarily mean that these compounds are not toxicants since they may bind to other receptors that perturb the toxicity pathways to induce systemic toxicity. In another word, an “active” response in a bioassay may relate directly to toxicity in animals, but an “inactive” result in a bioassay does not imply lack of toxicity in animals. To represent this scenario, we designed the novel *L* parameter as the second scoring criteria. **Figure 4** shows the relationship between *L* parameters and the true positive rates of the relevant bioassays for our target animal acute toxicity. It is notable that the true positive rates of the bioassays are all quite high (around 90%) when the *L* parameters are above 7 and in these instances, the number of FP cases is low (**Figure 4**). The *L* parameter well reflects the nature of our hypothesis. On the other hand, only using the *L* parameter to select bioassays may result in biased selections. For example, several bioassays with high *L* values that are located in area C of **Figure 3** all have high true positive rates but with few active responses. This condition may be due to chance when considering that the CCR is near to random. For this reason, we used a CCR>0.6, a typical cutoff used by many modeling approaches for model selection, to pre-select 95 bioassays out of the total of 555 bioassays. These 95 bioassays were then ranked by their *L* parameters. The top four assays, which are located in the area B of **Figure 3**, all have high true positive rates and the ratio of active responses in the testing result indicates that this correlation is not coincidental. The top ranked 47 bioassays (about 50% of the 95 bioassays) were selected to build the response profiles, in which all *L* parameters are larger than 6.4. All the 555 bioassays with their parameter information were listed in **Table S1** and the selected 47 bioassay were listed in **Table S2**.

In addition, we performed the Chi^2^ test [24] to examine the compound activity distributions between animal acute toxicity and the PubChem bioassays. A higher Chi^2^ value represents a higher relationship between bioassays and animal test. However, Chi^2^ test still cannot fully avoid biased conditions, similar to those shown in **Figure 3**. For example, the bioassay with AID 504847 is to identify inhibitors of the vitamin D receptor. It was ranked as one of the top 10 assays if we used Chi^2^ values as the criterion (Chi^2^ =  42.5 for this bioassay). The compounds tested by this bioassay have a large overlap with our acute toxicity database (727 compounds total) and that is the major reason that this bioassay has a high Chi^2^ value. Using our method, it was ranked #159. It shows somewhat relationship but does not rank as high as the most useful bioassays for animal acute toxicity (see section 3.4 for further discussion). Another example is the bioassay with AID 651741, which is to detect the agonists of the antioxidant response element (ARE) signaling pathway. This bioassay also shows high Chi^2^ value (40.8) but is ranked low in our study (#83). Although the Chi^2^ values show usefulness to indicate the importance of bioassays to animal toxicity, ranking by Chi^2^ values does not fit our hypothesis of systemic effect as described above. We listed all Chi^2^ values for the 555 bioassays in **Table S1** for comparison purposes.

### Classifying Compounds for their Animal Acute Toxicity using the Resulting Response Profiles

**Figure 5:**
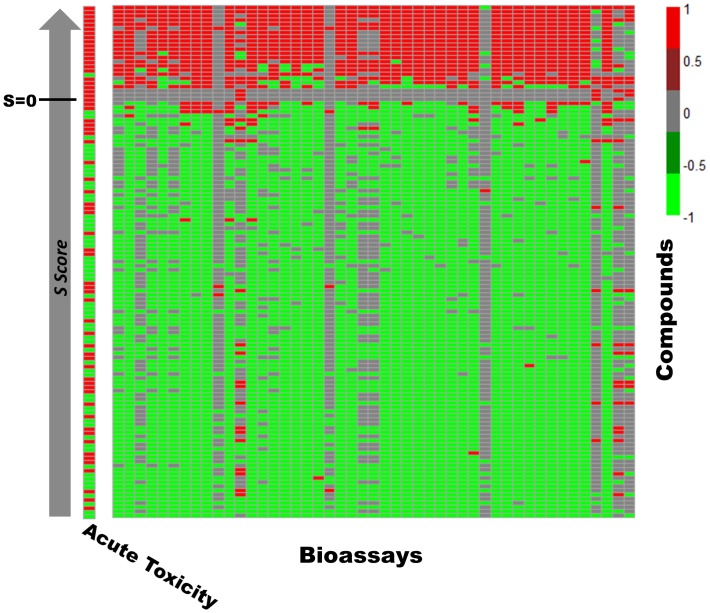
The reorganized heat map for the response profiles of 123 compounds against 47 bioassays. The left separated column shows the animal acute toxicity for each compound.

**Figure 6:**
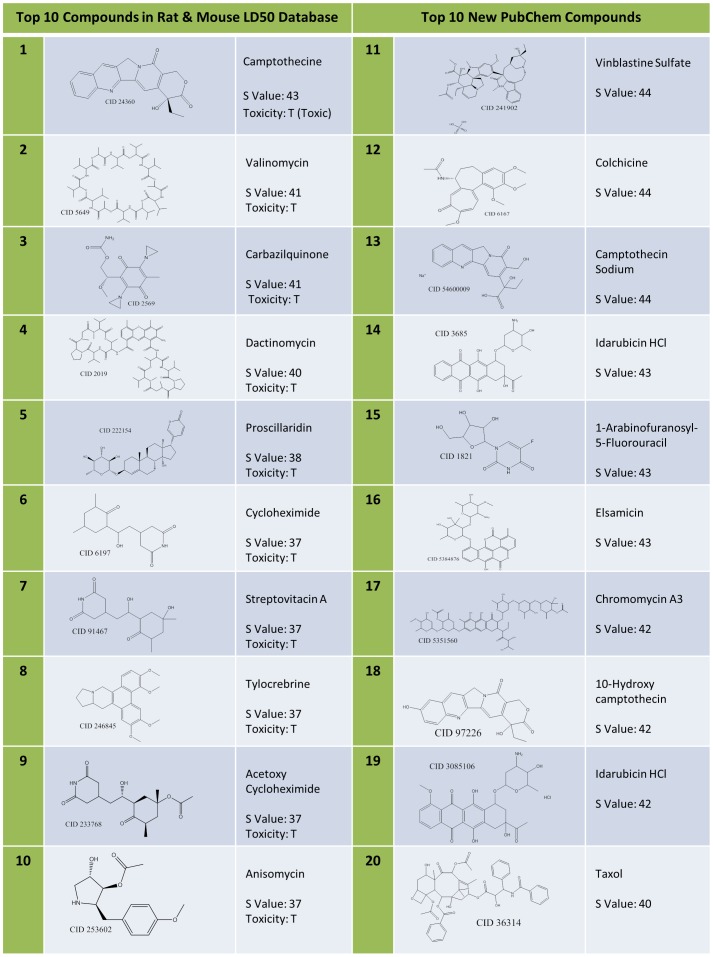
The list of the top ten compounds with the highest *S* values from our acute toxicity database (1–10) and from data mining of new PubChem compounds (11–20). The toxicity related excerpts and sources for the new PubChem compounds were listed in Table S3.

**Figure 7:**
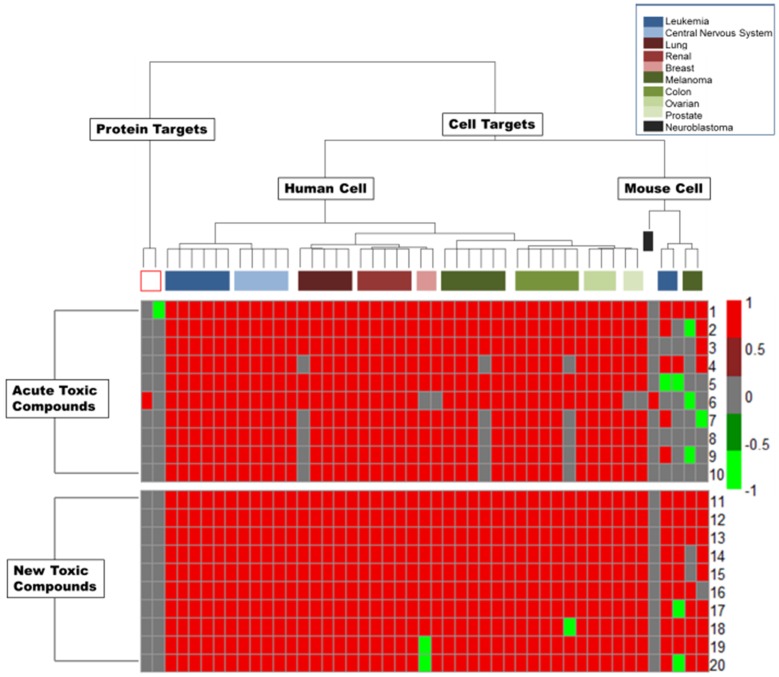
The heatmap of 10 top-ranked acute toxicity compounds (from 1–10) along with 10 top-ranked new PubChem compounds (from 11–20). All the compounds were ranked based on the *S* values of their biological responses in 47 bioassays identified in this study.

The resulting response profiles, consisting of 47 top ranked bioassays, were applied to classify the animal acute toxicity for the compounds of interest. It is well known that the bioassay data may contain experimental errors due to multiple reasons (using a single dose instead of multiple doses for testing, the purity of the samples, assay artifacts, etc.). Classification of a compound as toxic is relatively uncertain if it only shows active responses in one or two of bioassays we selected, although these bioassays show certain relationship to animal toxicity. On the other hand, if a compound has “missing data” or “inconclusive” results in many selected bioassays, it is not reasonable to consider it as non-toxic since it may show “active” results when testing against these bioassays in the future. Therefore, it is reasonable to define the following criteria when we use our selected bioassays to classify compounds: A compound will be selected for classification if: 1) three or more assays in our selected set of 47 bioassays have an active response for this compound; or 2) this compound has a reported response (either active or inactive) in more than 50% of the 47 assays (25 assays or more). There were 123 compounds selected from the Rat *LD_50_* dataset based on these criteria, and of these, 64 compounds were toxicants and 59 compounds were non-toxicants. The *S* scores of these 123 compounds were calculated. Using the *L* parameter to select bioassays and the *S* score to prioritize compounds, we could re-organize a portion of the original response profiles, as shown in **Figure 3**, into a new heatmap (**Figure 5**) illustrating the response profiles of our focused subset of 123 animal acute toxicants and non-toxicants. If we use the threshold of *S* = 0 to classify compounds, it is clear that the compounds with an *S* score above 0 are mostly toxic except one compound (CID 5405, terfenadine, an antihistamine used for the treatment of allergic conditions). It causes cardiotoxicity at high doses, but its major active metabolite does not [25]. However, those compounds with an *S* score below 0 contain both toxicants and non-toxicants, which also fit our hypothesis based on systemic effects, as described above. Based on this discovery, we propose that the compounds with larger *S* scores are potential toxicants by using the response profiles consisting of the top ranked bioassays in our study. **Figure 6** shows the molecular structures of 10 compounds with the highest *S* scores (Compound 1–10). Apparently they are not similar to each other if only considering their chemical structures. However, these compounds are indeed similar to each other based on their response profiles from the 47 bioassays (**Figure 7**).

These 47 top-ranked bioassays could also be used as a testing battery to prioritize new compounds for future animal testing. To prove their usefulness, we used these 47 bioassays to prioritize new PubChem compounds for their potential to be animal acute toxicants. There are 10,508 compounds that do not exist in our current Rat *LD_50_* acute toxicity data set, and each of them has an active response in at least one of the 47 bioassays. If we employ similar criteria to that described above, where a compound is required to have at least three active responses in the panel of 47 bioassays, there are 2,033 new PubChem compounds that are evaluated for potential animal acute toxicity. The maximum *S* score of these compounds is 44. The 10 compounds with the highest S score were also listed in **Figure 6** (Compound 11–20). And their response profiles were shown in **Figure 7** as a comparison to those of 10 compounds with the highest *S* score in our acute toxicity set. Compound **13** and **18** have similar chemical structures and are also comparable to compound **1** in our acute toxicity database (**Figure 6**). But most of the remaining compounds have different chemical structures, but similar response profiles.

We selected the top 32 new compounds, which all have an *S* score value larger than 41, and examined their toxicity potential by manually searching the literature and public sources. All 32 compounds, as listed in **Table S3**, have toxicity reports. Among them, six compounds have toxicity data in the Hazardous Substances Data Bank (http://toxnet.nlm.nih.gov/cgi-bin/sis/htmlgen?HSDB, Accessed June 1, 2013). They were experimentally identified to cause specific side effects in humans. Most of the other compounds (i.e., anthracycline antibiotics, camptothecin analogs, polypeptide antibiotics, etc.) were used as chemotherapeutic agents for cancers and were proved to be cytotoxic. Some of these compounds also have been tested in animals and show toxicity. For example, anthracycline antibiotics and their analogs are well-known for their genotoxicity and cardiotoxicity. Also, the anti-tubulin agents, vinblastine and colchicine, show genotoxicity in rat micronucleus test. The above analysis provides indirect but clear evidence of the applicability of our tool to prioritize potential toxicants for future animal testing.

### Potential Toxicity Mechanisms Relevant to the Selected Bioassays

**Table S1** lists all 555 PubChem bioassays used in this study, including the top 47 ranked bioassays in **Table S2** (all assays with CCR>0.6 for acute toxicity and ranked by their *L* values). It is noticeable that some bioassays have low correlations with animal acute toxicity (CCR<0.6), although the number of target compounds tested in these bioassay are relatively high. For example, non-cell-based receptor binding assays, such as BCL2 (AID 2129), tyrosyl-DNA phosphodiesterase (AID 485290), glutaminase (AID 624170 and 624146), etc., or the assays used to screen pathogens (i.e., *Mycobacterium tuberculosis* H37Rv (AID 1332), a notorious pathogen which increases resistance to antibiotics) apparently have no relationship with animal acute toxicity. Among all the assays having higher correlations with animal acute toxicity (CCR >0.6), their priority as potential screening alternatives could be ranked using the *L* value. The top 47 ranked bioassays selected in this study are mostly from the Developmental Therapeutics Program (DTP) of the National Cancer Institute (NCI). These assays belong to the NCI 60 human tumor cell lines used as an anticancer drug screen panel, called as NCI60 [21]. The protocols used in these assays could be classified by nine distinct tumor types: leukemia, colon, lung, CNS, renal, melanoma, ovarian, breast, and prostate (**Figure 7**). The screening used the sulforodamine B (SRB) assay, which is one of the most widely used methods for *in vitro* cytotoxicity screening, to identify compounds with growth-inhibitory or toxic effects on particular cell lines [26]. Hence, it is reasonable to assume that this assay might be relevant to *in vivo* acute toxicity [27]. The tumor mice model assays are used to estimate compound antitumor activity based on the percent of tumor shrinkage relative to the control, indicating the cytotoxicity of the compounds. Some of the NCI60 assays, using a human tumor xenograft mouse model, also have high rankings because of the high specificity and high coverage of non-toxic compounds (e.g., AID264).

The NCGC has deposited datasets for many bioassays potentially related to toxicity into PubChem, many of which were developed through collaboration with the NTP as part of the Tox21 project. Among them, there are 13 cell viability assays of various human primary cell lines [28]. Several of these were found to have a relatively high correlation (CCR>0.6) with animal acute toxicity (i.e., AID 426, AID 540 and AID 544). However, most of the NCGC cell viability assays were excluded from our top ranked 47 assays because of their relatively low *L* value. One possible reason for the scoring performance difference between the NCI60 assays and the NCGC cell viability assays could be related to the difference in definition of active compounds. In the NCI60 assays, a compound is considered to be active if the GI50 (concentration required for 50% inhibition of growth) is more potent at 1 µM or less. This is a stringent threshold used to define actives in bioassays. On the contrary, in the NCGC/NTP cell viability assays, an active compound could have an IC50 (concentration required for 50% of inhibition) ranging from 0.6 nM to 92 µM as long as the responses can be well fitted to the Hill equation. This method to define actives resulted in more false positives in the assays when compared with the animal toxicants. Interestingly, two other NCGC/NTP assays out of these 13 cell viability assays have higher rank in our study. These two cell-based assays primarily aimed at identifying compounds with specific inhibitory effects against either the D1 dopamine receptor (AID 488983) or retinoid-related orphan receptor gamma (AID 651802). We suspect that the true positives identified in these two assays could be cytotoxic and cytotoxicity is one of the potential artifacts that were not excluded when data were deposited into PubChem, which can also cause decreasing signal in assays.

## Conclusions

In this study, we developed a new data mining method to extract useful bioassays to generate response profiles for the evaluation of animal acute toxicity of organic compounds. Next, we developed a new scoring system to estimate the relevance between *in vitro* bioassay outcomes and the animal acute toxicity of 4,841 compounds. The top ranked 47 bioassays were considered to have potential *in vitro-in vivo* correlations, although the final number of compounds that could be evaluated due to sparse assay coverage was relatively small (N = 123). Most of these bioassays belong to the NCI60 human tumor cell lines applied as an anticancer drug screen panel. Moreover, we developed another scoring system to calculate the consensus *S* score for a compound based on its response profile. Compounds with higher *S* score are found to have higher toxicity potential. We tested the usefulness of the automatic response profiling workflow by prioritizing new PubChem compounds with toxicity potentials. The 32 compounds with the highest S scores were examined manually by literature searching and found to have positive results in various toxicity studies. The protocols and the whole profiling workflow developed in this study could be used to explore useful public bioassay data, especially the big data type resources, for compounds of interest, which are targeted by other complex bioactivities (i.e. other animal toxicities).

## Supporting Information

Table S1 All bioassays extracted from PubChem. These bioassays were ranked by the correlation to the animal acute toxicity using the tools described in this study.
(DOCX)

Table S2 The top-ranked 47 PubChem bioassays.
(DOCX)

Table S3 The top 32 new PubChem compounds ranked by *S* score. The compounds were prioritized using assay profiles which consists of 47 bioassays. Their toxicity excerpts were also listed.
(DOCX)

Protocol S1 The Perl scripts used in this study.
(ZIP)
