# Supplementary material for: Profiling Animal Toxicants by Automatically Mining Public Bioassay Data: A Big Data Approach for Computational Toxicology
Source: PLoS One. 2014 Jun 20;9(6):e99863. doi: 10.1371/journal.pone.0099863 (PMC4064997; doi:10.1371/journal.pone.0099863)
Supplement: Table S1 — All bioassays extracted from PubChem. These bioassays were ranked by the correlation to the animal acute toxicity using the tools described in this study. (DOCX) [file pone.0099863.s001.docx]

**Table S1**. The 555 bioassays extracted from the PubChem. These bioassays were ranked by the correlation to the animal acute toxicity using the tools described in this study.

| Rank | AID | L(A) | CCR | Chi Square |
| --- | --- | --- | --- | --- |
| 1 | 192 | 23.23 | 0.66 | 27.3 |
| 2 | 256 | 12.51 | 0.64 | 43.56 |
| 3 | 123 | 11.2 | 0.69 | 25.02 |
| 4 | 200 | 11 | 0.75 | 14.88 |
| 5 | 73 | 9.51 | 0.66 | 19.74 |
| 6 | 49 | 9.5 | 0.66 | 19.79 |
| 7 | 145 | 9.5 | 0.66 | 19.58 |
| 8 | 23 | 9.48 | 0.66 | 19.91 |
| 9 | 7 | 9.34 | 0.65 | 19.11 |
| 10 | 65 | 9.34 | 0.65 | 19.17 |
| 11 | 113 | 9.28 | 0.67 | 19.68 |
| 12 | 131 | 9.03 | 0.65 | 18.41 |
| 13 | 37 | 9.02 | 0.65 | 18.71 |
| 14 | 115 | 9 | 0.68 | 19.44 |
| 15 | 488983 | 9 | 0.61 | 10.26 |
| 16 | 109 | 8.71 | 0.65 | 17.8 |
| 17 | 137 | 8.68 | 0.65 | 17.65 |
| 18 | 19 | 8.56 | 0.64 | 17.11 |
| 19 | 45 | 8.41 | 0.64 | 16.8 |
| 20 | 53 | 8.41 | 0.64 | 16.8 |
| 21 | 33 | 8.22 | 0.63 | 16.15 |
| 22 | 5 | 8.21 | 0.64 | 16.19 |
| 23 | 47 | 8.11 | 0.64 | 16.19 |
| 24 | 61 | 8.08 | 0.68 | 10.32 |
| 25 | 69 | 8.08 | 0.68 | 10.32 |
| 26 | 25 | 8.05 | 0.65 | 16.29 |
| 27 | 77 | 8.04 | 0.64 | 16.01 |
| 28 | 651802 | 7.81 | 0.62 | 48.45 |
| 29 | 105 | 7.73 | 0.63 | 14.91 |
| 30 | 39 | 7.6 | 0.62 | 14.61 |
| 31 | 143 | 7.6 | 0.62 | 14.61 |
| 32 | 3 | 7.59 | 0.63 | 14.64 |
| 33 | 93 | 7.53 | 0.65 | 14.91 |
| 34 | 133 | 7.53 | 0.65 | 15.31 |
| 35 | 119 | 7.34 | 0.68 | 21.62 |
| 36 | 330 | 7.18 | 0.63 | 38.86 |
| 37 | 125 | 7.05 | 0.68 | 20.9 |
| 38 | 540 | 6.9 | 0.61 | 42.64 |
| 39 | 81 | 6.77 | 0.66 | 18.65 |
| 40 | 79 | 6.77 | 0.66 | 18.71 |
| 41 | 121 | 6.65 | 0.66 | 18.35 |
| 42 | 41 | 6.56 | 0.64 | 12.77 |
| 43 | 107 | 6.53 | 0.61 | 11.94 |
| 44 | 43 | 6.53 | 0.65 | 12.96 |
| 45 | 27 | 6.49 | 0.64 | 7.76 |
| 46 | 59 | 6.45 | 0.65 | 17.31 |
| 47 | 85 | 6.43 | 0.64 | 12.44 |
| 48 | 87 | 6.41 | 0.64 | 12.45 |
| 49 | 426 | 6.38 | 0.61 | 41.16 |
| 50 | 29 | 6.22 | 0.65 | 16.54 |
| 51 | 943 | 6 | 0.65 | 7.32 |
| 52 | 51 | 6 | 0.64 | 7.3 |
| 53 | 67 | 5.92 | 0.64 | 15.39 |
| 54 | 71 | 5.92 | 0.64 | 15.39 |
| 55 | 9 | 5.9 | 0.63 | 15.07 |
| 56 | 1 | 5.9 | 0.64 | 15.11 |
| 57 | 15 | 5.8 | 0.64 | 15.14 |
| 58 | 544 | 5.79 | 0.62 | 42.58 |
| 59 | 139 | 5.68 | 0.64 | 14.63 |
| 60 | 141 | 5.57 | 0.61 | 9.91 |
| 61 | 13 | 5.48 | 0.64 | 13.93 |
| 62 | 21 | 5.48 | 0.62 | 13.52 |
| 63 | 101 | 5.47 | 0.63 | 13.55 |
| 64 | 944 | 5.45 | 0.71 | 11.73 |
| 65 | 31 | 5.38 | 0.64 | 13.83 |
| 66 | 95 | 5.23 | 0.62 | 9.28 |
| 67 | 99 | 5 | 0.64 | 15.61 |
| 68 | 35 | 4.75 | 0.61 | 10.89 |
| 69 | 540276 | 4.41 | 0.62 | 21.12 |
| 70 | 328 | 4.35 | 0.7 | 31.86 |
| 71 | 103 | 4.34 | 0.61 | 9.96 |
| 72 | 91 | 3.89 | 0.62 | 8.57 |
| 73 | 1201 | 3.71 | 0.84 | 15.54 |
| 74 | 97 | 3.67 | 0.62 | 7.77 |
| 75 | 891 | 3.65 | 0.64 | 14.86 |
| 76 | 55 | 3.48 | 0.62 | 10.38 |
| 77 | 75 | 3.09 | 0.61 | 4.7 |
| 78 | 190 | 3 | 1 | 12 |
| 79 | 129 | 2.9 | 0.61 | 7.77 |
| 80 | 89 | 2.88 | 0.61 | 5.65 |
| 81 | 63 | 2.81 | 0.63 | 5.64 |
| 82 | 83 | 2.75 | 0.62 | 8.37 |
| 83 | 651741 | 2.68 | 0.65 | 40.8 |
| 84 | 1816 | 2.29 | 0.64 | 5.92 |
| 85 | 1877 | 2.2 | 0.6 | 4.19 |
| 86 | 1828 | 2.16 | 0.64 | 4.21 |
| 87 | 17 | 2.14 | 0.61 | 2.83 |
| 88 | 1883 | 2.05 | 0.66 | 6.47 |
| 89 | 651611 | 2 | 1 | 10 |
| 90 | 1815 | 1.95 | 0.61 | 3.95 |
| 91 | 2253 | 1.89 | 0.75 | 4.22 |
| 92 | 177 | 1.87 | 0.76 | 4.5 |
| 93 | 226 | 1.87 | 0.73 | 4.73 |
| 94 | 250 | 1.84 | 0.77 | 4.87 |
| 95 | 206 | 1.84 | 0.63 | 2.36 |
| 96 | 194 | 1.83 | 0.77 | 4.44 |
| 97 | 210 | 1.79 | 0.76 | 4 |
| 98 | 410 | 1.75 | 0.63 | 9.88 |
| 99 | 624379 | 1.69 | 0.92 | 6.35 |
| 100 | 2252 | 1.6 | 0.69 | 2.04 |
| 101 | 228 | 1.59 | 0.66 | 1.87 |
| 102 | 264 | 1.5 | 0.64 | 1.87 |
| 103 | 537735 | 1.5 | 0.64 | 1.4 |
| 104 | 377 | 1.43 | 0.61 | 3.74 |
| 105 | 463213 | 1.4 | 0.73 | 2.36 |
| 106 | 651982 | 1.18 | 0.62 | 0.85 |
| 107 | 159 | 1.17 | 0.61 | 0.78 |
| 108 | 1985 | 1.17 | 0.61 | 1.27 |
| 109 | 489028 | 1.15 | 0.6 | 1.21 |
| 110 | 232 | 1.13 | 0.71 | 1.64 |
| 111 | 288 | 1.09 | 0.7 | 1.59 |
| 112 | 435019 | 1 | 0.75 | 2.4 |
| 113 | 651613 | 1 | 0.75 | 4.44 |
| 114 | 651614 | 1 | 0.75 | 4.44 |
| 115 | 588794 | 0.9 | 0.95 | 4.95 |
| 116 | 336 | 0.89 | 0.94 | 4.44 |
| 117 | 624378 | 0.85 | 0.67 | 1.3 |
| 118 | 252 | 0.67 | 0.83 | 1.78 |
| 119 | 543 | 5.95 | 0.6 | 35.77 |
| 120 | 1886 | 2 | 0.6 | 3.77 |
| 121 | 1996 | 1.21 | 0.59 | 6.65 |
| 122 | 488745 | 2.58 | 0.59 | 4.06 |
| 123 | 248 | 12.41 | 0.59 | 60.1 |
| 124 | 504834 | 2.79 | 0.59 | 24.19 |
| 125 | 1882 | 2 | 0.59 | 3.21 |
| 126 | 1876 | 1.63 | 0.58 | 2.49 |
| 127 | 541 | 3.52 | 0.58 | 18.84 |
| 128 | 883 | 3.81 | 0.58 | 8.87 |
| 129 | 504332 | 2.91 | 0.58 | 27.21 |
| 130 | 175 | 4.51 | 0.58 | 44.46 |
| 131 | 1194 | 1.48 | 0.58 | 6.13 |
| 132 | 985 | 6.33 | 0.58 | 30.89 |
| 133 | 994 | 8.47 | 0.58 | 36.01 |
| 134 | 504444 | 14.48 | 0.58 | 38.11 |
| 135 | 161 | 4.69 | 0.58 | 42.94 |
| 136 | 155 | 4.18 | 0.58 | 39.48 |
| 137 | 270 | 1.44 | 0.58 | 1.38 |
| 138 | 427 | 10.78 | 0.57 | 36.06 |
| 139 | 157 | 4.91 | 0.57 | 43.13 |
| 140 | 981 | 5.01 | 0.57 | 23.93 |
| 141 | 435 | 6.42 | 0.57 | 27.92 |
| 142 | 651755 | 4.59 | 0.57 | 22.62 |
| 143 | 1900 | 1 | 0.57 | 0.78 |
| 144 | 1902 | 1 | 0.57 | 0.78 |
| 145 | 2384 | 1.08 | 0.57 | 0.8 |
| 146 | 488752 | 2.33 | 0.57 | 3.19 |
| 147 | 884 | 3.24 | 0.57 | 24.67 |
| 148 | 504652 | 5.69 | 0.57 | 18.39 |
| 149 | 972 | 5.65 | 0.57 | 24.91 |
| 150 | 504832 | 3.75 | 0.57 | 24.59 |
| 151 | 964 | 5.99 | 0.57 | 25.53 |
| 152 | 540256 | 3.02 | 0.57 | 9.72 |
| 153 | 968 | 5.91 | 0.57 | 24.22 |
| 154 | 165 | 5.03 | 0.57 | 39.17 |
| 155 | 167 | 3.87 | 0.57 | 32.16 |
| 156 | 961 | 5.41 | 0.57 | 23.41 |
| 157 | 651754 | 3.4 | 0.57 | 15.27 |
| 158 | 542 | 7.15 | 0.57 | 27.02 |
| 159 | 504847 | 5.74 | 0.56 | 42.54 |
| 160 | 970 | 5.25 | 0.56 | 21.66 |
| 161 | 921 | 5.09 | 0.56 | 20.79 |
| 162 | 987 | 4.79 | 0.56 | 19.91 |
| 163 | 521220 | 2 | 0.56 | 2.64 |
| 164 | 988 | 4.74 | 0.56 | 19.62 |
| 165 | 974 | 5.45 | 0.56 | 20.85 |
| 166 | 2330 | 2.72 | 0.56 | 6.84 |
| 167 | 488953 | 1.79 | 0.56 | 2.32 |
| 168 | 967 | 8.06 | 0.56 | 23.43 |
| 169 | 973 | 5.15 | 0.56 | 19.24 |
| 170 | 986 | 4.42 | 0.56 | 16.9 |
| 171 | 979 | 4.4 | 0.56 | 16.82 |
| 172 | 965 | 4.72 | 0.56 | 17.86 |
| 173 | 421 | 8.42 | 0.56 | 25.47 |
| 174 | 980 | 4.68 | 0.56 | 17.6 |
| 175 | 982 | 4.27 | 0.55 | 16.03 |
| 176 | 969 | 5.52 | 0.55 | 19.17 |
| 177 | 371 | 1.76 | 0.55 | 1.82 |
| 178 | 485346 | 3.98 | 0.55 | 15.35 |
| 179 | 971 | 4.45 | 0.55 | 15.61 |
| 180 | 975 | 4.75 | 0.55 | 16.39 |
| 181 | 978 | 4.3 | 0.55 | 14.84 |
| 182 | 899 | 2.08 | 0.55 | 3.34 |
| 183 | 588834 | 3.64 | 0.55 | 14.49 |
| 184 | 804 | 4.52 | 0.55 | 6.24 |
| 185 | 545 | 4.41 | 0.55 | 13.96 |
| 186 | 602229 | 28.36 | 0.55 | 29.22 |
| 187 | 960 | 3.78 | 0.55 | 11.54 |
| 188 | 1030 | 1.78 | 0.55 | 8.98 |
| 189 | 433 | 8.66 | 0.55 | 21.58 |
| 190 | 1460 | 2.92 | 0.55 | 10.09 |
| 191 | 624296 | 3.87 | 0.54 | 18.48 |
| 192 | 984 | 3.93 | 0.54 | 12.23 |
| 193 | 546 | 6.72 | 0.54 | 17.88 |
| 194 | 2717 | 4.43 | 0.54 | 12.97 |
| 195 | 2667 | 2.45 | 0.54 | 2.87 |
| 196 | 966 | 4.41 | 0.54 | 12.54 |
| 197 | 655 | 4.99 | 0.54 | 14.16 |
| 198 | 915 | 2.05 | 0.54 | 2.8 |
| 199 | 434 | 7.96 | 0.54 | 18.73 |
| 200 | 588850 | 5.04 | 0.54 | 16.21 |
| 201 | 977 | 4.44 | 0.54 | 12.67 |
| 202 | 818 | 3.01 | 0.54 | 4.14 |
| 203 | 983 | 3.94 | 0.54 | 11.07 |
| 204 | 993 | 4.83 | 0.54 | 13.5 |
| 205 | 588511 | 4.54 | 0.54 | 10.2 |
| 206 | 2551 | 5.33 | 0.54 | 29.63 |
| 207 | 948 | 4.7 | 0.54 | 12.98 |
| 208 | 2546 | 6.27 | 0.54 | 31.99 |
| 209 | 1578 | 5.63 | 0.54 | 8.51 |
| 210 | 1813 | 3.74 | 0.54 | 7.28 |
| 211 | 493107 | 1.71 | 0.54 | 1.58 |
| 212 | 1490 | 2.61 | 0.54 | 11.69 |
| 213 | 485298 | 5.4 | 0.54 | 13.31 |
| 214 | 588405 | 4.01 | 0.54 | 12.4 |
| 215 | 894 | 3.69 | 0.54 | 17.33 |
| 216 | 651758 | 2.02 | 0.54 | 1.67 |
| 217 | 651743 | 4.45 | 0.54 | 18.34 |
| 218 | 431 | 1.64 | 0.54 | 1.32 |
| 219 | 2313 | 3.71 | 0.54 | 5.19 |
| 220 | 504333 | 2.2 | 0.54 | 5.23 |
| 221 | 463190 | 4.47 | 0.53 | 11.19 |
| 222 | 912 | 4.78 | 0.53 | 13.46 |
| 223 | 463097 | 1.36 | 0.53 | 0.78 |
| 224 | 588537 | 5.26 | 0.53 | 13.47 |
| 225 | 651820 | 3.09 | 0.53 | 8.16 |
| 226 | 504865 | 2.89 | 0.53 | 7.71 |
| 227 | 463189 | 2.08 | 0.53 | 2.18 |
| 228 | 463212 | 8.95 | 0.53 | 14.52 |
| 229 | 588513 | 3.94 | 0.53 | 12.23 |
| 230 | 955 | 5.09 | 0.53 | 11.1 |
| 231 | 588354 | 9.78 | 0.53 | 16.23 |
| 232 | 652054 | 3.12 | 0.53 | 8.53 |
| 233 | 2275 | 2.67 | 0.53 | 3.79 |
| 234 | 989 | 3.78 | 0.53 | 8.16 |
| 235 | 463195 | 5.97 | 0.53 | 11.6 |
| 236 | 976 | 3.76 | 0.53 | 8.09 |
| 237 | 963 | 4.22 | 0.53 | 9.14 |
| 238 | 1362 | 3.62 | 0.53 | 4.44 |
| 239 | 588855 | 5.1 | 0.53 | 11.44 |
| 240 | 946 | 3.66 | 0.53 | 7.72 |
| 241 | 488949 | 1.57 | 0.53 | 1.26 |
| 242 | 624032 | 1.82 | 0.53 | 2.81 |
| 243 | 485344 | 1.97 | 0.53 | 3.8 |
| 244 | 2825 | 4.47 | 0.53 | 9.29 |
| 245 | 435005 | 3.85 | 0.53 | 7.2 |
| 246 | 651661 | 8.47 | 0.53 | 13.63 |
| 247 | 962 | 3.56 | 0.53 | 7.41 |
| 248 | 306 | 0.74 | 0.53 | 0.03 |
| 249 | 444 | 1.88 | 0.53 | 1.79 |
| 250 | 1285 | 2.44 | 0.53 | 2.69 |
| 251 | 588352 | 8.7 | 0.53 | 14.02 |
| 252 | 485314 | 3.32 | 0.53 | 4.94 |
| 253 | 488899 | 3.75 | 0.53 | 8.3 |
| 254 | 902 | 4.85 | 0.53 | 14.07 |
| 255 | 588674 | 5.76 | 0.53 | 11.05 |
| 256 | 504558 | 2.36 | 0.53 | 5.22 |
| 257 | 598 | 1.45 | 0.52 | 0.79 |
| 258 | 2716 | 3.78 | 0.52 | 6.99 |
| 259 | 504582 | 3.49 | 0.52 | 7.11 |
| 260 | 463082 | 3.9 | 0.52 | 7.3 |
| 261 | 588533 | 4.75 | 0.52 | 11.16 |
| 262 | 2147 | 2.08 | 0.52 | 3.3 |
| 263 | 602449 | 3.46 | 0.52 | 7.02 |
| 264 | 504621 | 3.68 | 0.52 | 6.7 |
| 265 | 624352 | 5.05 | 0.52 | 9.07 |
| 266 | 596 | 2.66 | 0.52 | 6.34 |
| 267 | 504660 | 2.85 | 0.52 | 4.84 |
| 268 | 430 | 1.31 | 0.52 | 0.54 |
| 269 | 947 | 3.06 | 0.52 | 4.42 |
| 270 | 588397 | 0.88 | 0.52 | 0.05 |
| 271 | 1672 | 3.65 | 0.52 | 4.44 |
| 272 | 1063 | 1.46 | 0.52 | 0.87 |
| 273 | 588856 | 3.31 | 0.52 | 6.5 |
| 274 | 624354 | 3.79 | 0.52 | 6.99 |
| 275 | 651610 | 2.55 | 0.52 | 3.08 |
| 276 | 588852 | 6.52 | 0.52 | 9.69 |
| 277 | 504467 | 2.84 | 0.52 | 3.95 |
| 278 | 651635 | 3.62 | 0.52 | 6.52 |
| 279 | 588342 | 1.77 | 0.52 | 1.94 |
| 280 | 588526 | 15.93 | 0.52 | 16.07 |
| 281 | 624030 | 2.6 | 0.52 | 3.83 |
| 282 | 624146 | 1.18 | 0.52 | 0.47 |
| 283 | 651751 | 1.63 | 0.52 | 2.68 |
| 284 | 588413 | 3.14 | 0.52 | 4.95 |
| 285 | 1456 | 2.32 | 0.52 | 2.19 |
| 286 | 485270 | 1.95 | 0.52 | 2.45 |
| 287 | 1463 | 3.98 | 0.52 | 5.15 |
| 288 | 588546 | 3.21 | 0.52 | 5.87 |
| 289 | 624125 | 2.62 | 0.52 | 3.87 |
| 290 | 2071 | 2.44 | 0.51 | 2.35 |
| 291 | 886 | 1.59 | 0.51 | 1.85 |
| 292 | 2057 | 1.68 | 0.51 | 1.62 |
| 293 | 1688 | 2.34 | 0.51 | 3.12 |
| 294 | 887 | 2.07 | 0.51 | 3.2 |
| 295 | 449762 | 2.94 | 0.51 | 3.57 |
| 296 | 924 | 3.08 | 0.51 | 5.13 |
| 297 | 588378 | 1.07 | 0.51 | 0.42 |
| 298 | 463079 | 2.97 | 0.51 | 3.63 |
| 299 | 624031 | 3.14 | 0.51 | 3.37 |
| 300 | 2417 | 1.17 | 0.51 | 0.27 |
| 301 | 540303 | 2.14 | 0.51 | 2.39 |
| 302 | 504770 | 1.62 | 0.51 | 0.96 |
| 303 | 595 | 2.63 | 0.51 | 3.59 |
| 304 | 588590 | 1.82 | 0.51 | 1.85 |
| 305 | 662 | 2.92 | 0.51 | 3.75 |
| 306 | 624040 | 2.91 | 0.51 | 3.5 |
| 307 | 2517 | 2.25 | 0.51 | 4.01 |
| 308 | 1531 | 1.79 | 0.51 | 1.3 |
| 309 | 504845 | 3.85 | 0.51 | 4.72 |
| 310 | 1468 | 1.96 | 0.51 | 1.91 |
| 311 | 893 | 1.32 | 0.51 | 0.93 |
| 312 | 493033 | 3.65 | 0.51 | 3.67 |
| 313 | 1446 | 1.26 | 0.51 | 0.44 |
| 314 | 1481 | 1.26 | 0.51 | 0.44 |
| 315 | 485297 | 2.35 | 0.51 | 2.46 |
| 316 | 1021 | 1.28 | 0.51 | 0.46 |
| 317 | 624417 | 1.89 | 0.51 | 1.6 |
| 318 | 938 | 1.81 | 0.51 | 1.64 |
| 319 | 651777 | 2.11 | 0.51 | 2.72 |
| 320 | 1188 | 1.01 | 0.51 | 0.88 |
| 321 | 652048 | 2.14 | 0.51 | 2.95 |
| 322 | 652051 | 2.14 | 0.51 | 2.95 |
| 323 | 624297 | 1.75 | 0.51 | 1.84 |
| 324 | 1825 | 1.28 | 0.51 | 0.46 |
| 325 | 2599 | 1.79 | 0.51 | 1.4 |
| 326 | 624169 | 3.27 | 0.51 | 3.55 |
| 327 | 624256 | 2.38 | 0.51 | 2.24 |
| 328 | 449728 | 1.27 | 0.51 | 0.52 |
| 329 | 485317 | 1.22 | 0.51 | 0.37 |
| 330 | 2751 | 2.23 | 0.51 | 1.99 |
| 331 | 2629 | 1.22 | 0.51 | 0.4 |
| 332 | 504327 | 2.08 | 0.51 | 2.47 |
| 333 | 588506 | 0.98 | 0.51 | 0.07 |
| 334 | 2642 | 1.37 | 0.51 | 0.57 |
| 335 | 485364 | 1.2 | 0.51 | 0.34 |
| 336 | 2549 | 1.97 | 0.51 | 2.47 |
| 337 | 588591 | 1.71 | 0.51 | 1.23 |
| 338 | 624349 | 1.39 | 0.51 | 0.68 |
| 339 | 1662 | 1.12 | 0.51 | 0.19 |
| 340 | 2540 | 1.68 | 0.51 | 1.04 |
| 341 | 2235 | 1.62 | 0.51 | 0.94 |
| 342 | 651778 | 2.74 | 0.51 | 2.84 |
| 343 | 880 | 1.53 | 0.51 | 1.17 |
| 344 | 1974 | 1.21 | 0.51 | 0.32 |
| 345 | 1885 | 1.24 | 0.51 | 0.4 |
| 346 | 995 | 1.96 | 0.51 | 1.51 |
| 347 | 2239 | 1.22 | 0.51 | 0.38 |
| 348 | 1476 | 1.72 | 0.51 | 1.23 |
| 349 | 588579 | 1.43 | 0.51 | 0.87 |
| 350 | 881 | 1.5 | 0.5 | 0.78 |
| 351 | 588795 | 1.3 | 0.5 | 0.46 |
| 352 | 1479 | 2.22 | 0.5 | 2.23 |
| 353 | 489030 | 1.12 | 0.5 | 0.17 |
| 354 | 624044 | 1.05 | 0.5 | 0.1 |
| 355 | 588453 | 1.11 | 0.5 | 0.16 |
| 356 | 2732 | 0.93 | 0.5 | 0.04 |
| 357 | 1947 | 0.91 | 0.5 | 0.01 |
| 358 | 1458 | 1.01 | 0.5 | 0.06 |
| 359 | 1471 | 0.96 | 0.5 | 0.05 |
| 360 | 1422 | 0.8 | 0.5 | 0.01 |
| 361 | 588516 | 0.77 | 0.5 | 0 |
| 362 | 184 | 0.5 | 0.5 | NA |
| 363 | 238 | 0.5 | 0.5 | NA |
| 364 | 260 | 0.5 | 0.5 | 0 |
| 365 | 918 | 0.5 | 0.5 | NA |
| 366 | 1195 | 0.99 | 0.5 | NA |
| 367 | 1596 | 0.8 | 0.5 | NA |
| 368 | 1606 | 0.8 | 0.5 | NA |
| 369 | 1608 | 0.8 | 0.5 | NA |
| 370 | 1609 | 0.8 | 0.5 | NA |
| 371 | 1610 | 0.8 | 0.5 | NA |
| 372 | 1811 | 0.97 | 0.5 | NA |
| 373 | 1851 | 0.96 | 0.5 | NA |
| 374 | 1988 | 0.67 | 0.5 | NA |
| 375 | 2061 | 0.5 | 0.5 | NA |
| 376 | 2062 | 0.94 | 0.5 | NA |
| 377 | 2063 | 0.75 | 0.5 | NA |
| 378 | 2306 | 0 | 0.5 | NA |
| 379 | 2701 | 0.67 | 0.5 | 0 |
| 380 | 7783 | 0.92 | 0.5 | NA |
| 381 | 19006 | 0.75 | 0.5 | NA |
| 382 | 19424 | 0.91 | 0.5 | NA |
| 383 | 19468 | 0.67 | 0.5 | NA |
| 384 | 22293 | 0.93 | 0.5 | NA |
| 385 | 23271 | 0.75 | 0.5 | NA |
| 386 | 23734 | 0.83 | 0.5 | NA |
| 387 | 26304 | 0.93 | 0.5 | NA |
| 388 | 26362 | 0.83 | 0.5 | NA |
| 389 | 26811 | 0.83 | 0.5 | NA |
| 390 | 27167 | 0.95 | 0.5 | NA |
| 391 | 28681 | 0.95 | 0.5 | NA |
| 392 | 29359 | 0.95 | 0.5 | NA |
| 393 | 54923 | 0.67 | 0.5 | NA |
| 394 | 161281 | 0.75 | 0.5 | NA |
| 395 | 205267 | 0.67 | 0.5 | NA |
| 396 | 240820 | 0.5 | 0.5 | NA |
| 397 | 241172 | 0.88 | 0.5 | NA |
| 398 | 241174 | 0.88 | 0.5 | NA |
| 399 | 311367 | 0.95 | 0.5 | NA |
| 400 | 311934 | 0.83 | 0.5 | NA |
| 401 | 311935 | 0.83 | 0.5 | NA |
| 402 | 346025 | 0.98 | 0.5 | NA |
| 403 | 350218 | 0.86 | 0.5 | NA |
| 404 | 350219 | 0.86 | 0.5 | NA |
| 405 | 350220 | 0.86 | 0.5 | NA |
| 406 | 360149 | 0.86 | 0.5 | NA |
| 407 | 361985 | 0.8 | 0.5 | NA |
| 408 | 361986 | 0.8 | 0.5 | NA |
| 409 | 386625 | 0.5 | 0.5 | NA |
| 410 | 397743 | 0.67 | 0.5 | NA |
| 411 | 407366 | 0.67 | 0.5 | NA |
| 412 | 435006 | 0.75 | 0.5 | NA |
| 413 | 444054 | 0.96 | 0.5 | NA |
| 414 | 444055 | 0.96 | 0.5 | NA |
| 415 | 444056 | 0.96 | 0.5 | NA |
| 416 | 444057 | 0.96 | 0.5 | NA |
| 417 | 449703 | 0 | 0.5 | NA |
| 418 | 449704 | 0 | 0.5 | NA |
| 419 | 449706 | 0 | 0.5 | NA |
| 420 | 449756 | 0.8 | 0.5 | NA |
| 421 | 449764 | 0.67 | 0.5 | NA |
| 422 | 455986 | 0.94 | 0.5 | NA |
| 423 | 463074 | 0.67 | 0.5 | NA |
| 424 | 463215 | 0.67 | 0.5 | NA |
| 425 | 463229 | 0.8 | 0.5 | NA |
| 426 | 477295 | 0 | 0.5 | NA |
| 427 | 493017 | 0.5 | 0.5 | NA |
| 428 | 504749 | 0.97 | 0.5 | NA |
| 429 | 540211 | 0.98 | 0.5 | NA |
| 430 | 540234 | 0.5 | 0.5 | NA |
| 431 | 540237 | 0.67 | 0.5 | NA |
| 432 | 540268 | 0.67 | 0.5 | NA |
| 433 | 540270 | 0.67 | 0.5 | NA |
| 434 | 547621 | 0 | 0.5 | NA |
| 435 | 547622 | 0.5 | 0.5 | NA |
| 436 | 567091 | 0.95 | 0.5 | NA |
| 437 | 588214 | 0.9 | 0.5 | NA |
| 438 | 588215 | 0.86 | 0.5 | NA |
| 439 | 588216 | 0.92 | 0.5 | NA |
| 440 | 588217 | 0.9 | 0.5 | NA |
| 441 | 588218 | 0.75 | 0.5 | NA |
| 442 | 588219 | 0.83 | 0.5 | NA |
| 443 | 588812 | 0.93 | 0.5 | NA |
| 444 | 588813 | 0.94 | 0.5 | NA |
| 445 | 592681 | 0.94 | 0.5 | NA |
| 446 | 604020 | 0.75 | 0.5 | NA |
| 447 | 624180 | 0 | 0.5 | NA |
| 448 | 624190 | 0 | 0.5 | NA |
| 449 | 624192 | 0 | 0.5 | NA |
| 450 | 624209 | 0 | 0.5 | NA |
| 451 | 624223 | 0 | 0.5 | NA |
| 452 | 625146 | 0.86 | 0.5 | NA |
| 453 | 625151 | 0.5 | 0.5 | NA |
| 454 | 625152 | 0.5 | 0.5 | NA |
| 455 | 625153 | 0.5 | 0.5 | NA |
| 456 | 625154 | 0.5 | 0.5 | NA |
| 457 | 625155 | 0.5 | 0.5 | NA |
| 458 | 625162 | 0.75 | 0.5 | NA |
| 459 | 625163 | 0.67 | 0.5 | NA |
| 460 | 625171 | 0.67 | 0.5 | NA |
| 461 | 625181 | 0 | 0.5 | NA |
| 462 | 625184 | 0.75 | 0.5 | NA |
| 463 | 625185 | 0.75 | 0.5 | NA |
| 464 | 625190 | 0.67 | 0.5 | NA |
| 465 | 625191 | 0 | 0.5 | NA |
| 466 | 625192 | 0.8 | 0.5 | NA |
| 467 | 625196 | 0.67 | 0.5 | NA |
| 468 | 625198 | 0.75 | 0.5 | NA |
| 469 | 625199 | 0.75 | 0.5 | NA |
| 470 | 625200 | 0.75 | 0.5 | NA |
| 471 | 625201 | 0.86 | 0.5 | NA |
| 472 | 625202 | 0.8 | 0.5 | NA |
| 473 | 625203 | 0.75 | 0.5 | NA |
| 474 | 625207 | 0.89 | 0.5 | NA |
| 475 | 625215 | 0.8 | 0.5 | NA |
| 476 | 625216 | 0.75 | 0.5 | NA |
| 477 | 625217 | 0.86 | 0.5 | NA |
| 478 | 625218 | 0.83 | 0.5 | NA |
| 479 | 625221 | 0.75 | 0.5 | NA |
| 480 | 625222 | 0.88 | 0.5 | NA |
| 481 | 625223 | 0.75 | 0.5 | NA |
| 482 | 625224 | 0.75 | 0.5 | NA |
| 483 | 625225 | 0.83 | 0.5 | NA |
| 484 | 625227 | 0.75 | 0.5 | NA |
| 485 | 625228 | 0.8 | 0.5 | NA |
| 486 | 625229 | 0.5 | 0.5 | NA |
| 487 | 625234 | 0.75 | 0.5 | NA |
| 488 | 625243 | 0.8 | 0.5 | NA |
| 489 | 625244 | 0.5 | 0.5 | NA |
| 490 | 625245 | 0.75 | 0.5 | NA |
| 491 | 625247 | 0.67 | 0.5 | NA |
| 492 | 625248 | 0.75 | 0.5 | NA |
| 493 | 625249 | 0.8 | 0.5 | NA |
| 494 | 625251 | 0.67 | 0.5 | NA |
| 495 | 625252 | 0.67 | 0.5 | NA |
| 496 | 625253 | 0.83 | 0.5 | NA |
| 497 | 625254 | 0.83 | 0.5 | NA |
| 498 | 625256 | 0.83 | 0.5 | NA |
| 499 | 625269 | 0.67 | 0.5 | NA |
| 500 | 625270 | 0.75 | 0.5 | NA |
| 501 | 625272 | 0 | 0.5 | NA |
| 502 | 651838 | 0.98 | 0.5 | NA |
| 503 | 588544 | 0.85 | 0.5 | 0 |
| 504 | 1863 | 0.87 | 0.5 | 0 |
| 505 | 485290 | 0.67 | 0.5 | 0.09 |
| 506 | 435030 | 0.89 | 0.5 | 0.01 |
| 507 | 504339 | 0.91 | 0.5 | 0.02 |
| 508 | 2472 | 0.69 | 0.5 | 0.1 |
| 509 | 602346 | 0.61 | 0.5 | 0.18 |
| 510 | 588514 | 0.7 | 0.5 | 0.18 |
| 511 | 504803 | 0.36 | 0.5 | 0.63 |
| 512 | 504690 | 0.35 | 0.5 | 0.68 |
| 513 | 1981 | 0.87 | 0.49 | 0 |
| 514 | 2401 | 0.87 | 0.49 | 0 |
| 515 | 624170 | 0 | 0.49 | 2.88 |
| 516 | 2451 | 0.4 | 0.49 | 1.03 |
| 517 | 1814 | 0.54 | 0.49 | 0.34 |
| 518 | 651602 | 0.62 | 0.49 | 0.53 |
| 519 | 602440 | 0.54 | 0.49 | 0.69 |
| 520 | 1022 | 0.53 | 0.49 | 0.3 |
| 521 | 1850 | 0.69 | 0.49 | 0.33 |
| 522 | 504766 | 0.43 | 0.49 | 0.94 |
| 523 | 588458 | 0.27 | 0.49 | 1.38 |
| 524 | 1903 | 0 | 0.49 | 1.79 |
| 525 | 1332 | 0.83 | 0.49 | 0.1 |
| 526 | 2129 | 0.27 | 0.49 | 1.39 |
| 527 | 492953 | 0.24 | 0.49 | 1.77 |
| 528 | 540317 | 0 | 0.49 | 3 |
| 529 | 2315 | 0.74 | 0.49 | 0.44 |
| 530 | 651640 | 0.53 | 0.49 | 1.14 |
| 531 | 588726 | 0 | 0.49 | 3.75 |
| 532 | 589 | 0.72 | 0.48 | 0.47 |
| 533 | 2314 | 0.62 | 0.48 | 1.41 |
| 534 | 590 | 0.69 | 0.48 | 0.62 |
| 535 | 1208 | 0.9 | 0.48 | 0.52 |
| 536 | 1199 | 0.87 | 0.47 | 0.59 |
| 537 | 1915 | 0.8 | 0.47 | 0.07 |
| 538 | 1189 | 0.85 | 0.46 | 1.71 |
| 539 | 1205 | 0.81 | 0.45 | 1.98 |
| 540 | 588824 | 0 | 0.45 | NA |
| 541 | 180 | 0.47 | 0.44 | 0.44 |
| 542 | 463218 | 0.44 | 0.44 | 0.14 |
| 543 | 537733 | 0.67 | 0.43 | 0.31 |
| 544 | 488955 | 0.77 | 0.42 | 2.12 |
| 545 | 220 | 0.62 | 0.41 | 1.06 |
| 546 | 504607 | 0.54 | 0.41 | 0.43 |
| 547 | 258 | 0.39 | 0.39 | 0.27 |
| 548 | 212 | 0.55 | 0.39 | 1.36 |
| 549 | 504668 | 0.57 | 0.39 | 0.83 |
| 550 | 1204 | 0.24 | 0.39 | 2.44 |
| 551 | 1191 | 0.31 | 0.35 | 1.72 |
| 552 | 651983 | 0.43 | 0.32 | 1.34 |
| 553 | 488942 | 0.5 | 0.29 | 4.02 |
| 554 | 488956 | 0.36 | 0.25 | 4.5 |
| 555 | 651984 | 0.31 | 0.17 | 9.9 |
