# Supplementary material for: Profiling Animal Toxicants by Automatically Mining Public Bioassay Data: A Big Data Approach for Computational Toxicology
Source: PLoS One. 2014 Jun 20;9(6):e99863. doi: 10.1371/journal.pone.0099863 (PMC4064997; doi:10.1371/journal.pone.0099863)
Supplement: Table S2 — The top-ranked 47 PubChem bioassays. (DOCX) [file pone.0099863.s002.docx]

**Table S2**: The top-ranked 47 PubChem bioassays.

| PubChem Aid | AOM* | Source | Target | Description |
| --- | --- | --- | --- | --- |
| 192 | other | DTP/NCI | Cell | NCI In Vivo Anticancer Drug Screen. Data for tumor model B16 Melanoma (intraperitoneal) in B6D2F1 (BDF1) mice |
| 256 | other | DTP/NCI | Cell | NCI In Vivo Anticancer Drug Screen. Data for tumor model L1210 Leukemia (intraperitoneal) in CD2F1 (CDF1) mice |
| 123 | confirmatory | DTP/NCI | Cell | NCI human tumor cell line growth inhibition assay. Data for the MOLT-4 Leukemia cell line |
| 200 | other | DTP/NCI | Cell | NCI In Vivo Anticancer Drug Screen. Data for tumor model B16 Melanoma (intraperitoneal) in B6C3F1 mice |
| 73 | confirmatory | DTP/NCI | Cell | NCI human tumor cell line growth inhibition assay. Data for the KM12 Colon cell line |
| 49 | confirmatory | DTP/NCI | Cell | NCI human tumor cell line growth inhibition assay. Data for the SF-539 Central Nervous System cell line |
| 145 | confirmatory | DTP/NCI | Cell | NCI human tumor cell line growth inhibition assay. Data for the SN12C Renal cell line |
| 23 | confirmatory | DTP/NCI | Cell | NCI human tumor cell line growth inhibition assay. Data for the LOX IMVI Melanoma cell line |
| 7 | confirmatory | DTP/NCI | Cell | NCI human tumor cell line growth inhibition assay. Data for the NCI-H460 Non-Small Cell Lung cell line |
| 65 | confirmatory | DTP/NCI | Cell | NCI human tumor cell line growth inhibition assay. Data for the HT29 Colon cell line |
| 113 | confirmatory | DTP/NCI | Cell | NCI human tumor cell line growth inhibition assay. Data for the RPMI-8226 Leukemia cell line |
| 131 | confirmatory | DTP/NCI | Cell | NCI human tumor cell line growth inhibition assay. Data for the CAKI-1 Renal cell line |
| 37 | confirmatory | DTP/NCI | Cell | NCI human tumor cell line growth inhibition assay. Data for the SK-MEL-5 Melanoma cell line |
| 488983 | confirmatory | NCGC | D(1A) dopamine receptor [Homo sapiens] | HTS Assay for Allosteric Agonists of the Human D1 Dopamine Receptor: Primary Screen for Antagonists |
| 115 | confirmatory | DTP/NCI | Cell | NCI human tumor cell line growth inhibition assay. Data for the SR Leukemia cell line |
| 109 | confirmatory | DTP/NCI | Cell | NCI human tumor cell line growth inhibition assay. Data for the OVCAR-8 Ovarian cell line |
| 137 | confirmatory | DTP/NCI | Cell | NCI human tumor cell line growth inhibition assay. Data for the 786-0 Renal cell line |
| 19 | confirmatory | DTP/NCI | Cell | NCI human tumor cell line growth inhibition assay. Data for the A549/ATCC Non-Small Cell Lung cell line |
| 45 | confirmatory | DTP/NCI | Cell | NCI human tumor cell line growth inhibition assay. Data for the SF-268 Central Nervous System cell line |
| 53 | confirmatory | DTP/NCI | Cell | NCI human tumor cell line growth inhibition assay. Data for the SNB-19 Central Nervous System cell line |
| 33 | confirmatory | DTP/NCI | Cell | NCI human tumor cell line growth inhibition assay. Data for the UACC-257 Melanoma cell line |
| 5 | confirmatory | DTP/NCI | Cell | NCI human tumor cell line growth inhibition assay. Data for the NCI-H322M Non-Small Cell Lung cell line |
| 47 | confirmatory | DTP/NCI | Cell | NCI human tumor cell line growth inhibition assay. Data for the SF-295 Central Nervous System cell line |
| 69 | confirmatory | DTP/NCI | Cell | NCI human tumor cell line growth inhibition assay. Data for the DLD-1 Colon cell line |
| 61 | confirmatory | DTP/NCI | Cell | NCI human tumor cell line growth inhibition assay. Data for the DMS 273 Small Cell Lung cell line |
| 25 | confirmatory | DTP/NCI | Cell | NCI human tumor cell line growth inhibition assay. Data for the M14 Melanoma cell line |
| 77 | confirmatory | DTP/NCI | Cell | NCI human tumor cell line growth inhibition assay. Data for the HCC-2998 Colon cell line |
| 651802 | confirmatory | NCGC | Nuclear receptor ROR-gamma [Homo sapiens] | qHTS assay for small molecule antagonists of the retinoid-related orphan receptor gamma (ROR-gamma) signaling pathway |
| 105 | confirmatory | DTP/NCI | Cell | NCI human tumor cell line growth inhibition assay. Data for the OVCAR-4 Ovarian cell line |
| 39 | confirmatory | DTP/NCI | Cell | NCI human tumor cell line growth inhibition assay. Data for the SK-MEL-28 Melanoma cell line |
| 143 | confirmatory | DTP/NCI | Cell | NCI human tumor cell line growth inhibition assay. Data for the UO-31 Renal cell line |
| 3 | confirmatory | DTP/NCI | Cell | NCI human tumor cell line growth inhibition assay. Data for the NCI-H226 Non-Small Cell Lung cell line |
| 93 | confirmatory | DTP/NCI | Cell | NCI human tumor cell line growth inhibition assay. Data for the NCI/ADR-RES Breast cell line |
| 133 | confirmatory | DTP/NCI | Cell | NCI human tumor cell line growth inhibition assay. Data for the RXF 393 Renal cell line |
| 119 | confirmatory | DTP/NCI | Cell | NCI human tumor cell line growth inhibition assay. Data for the CCRF-CEM Leukemia cell line |
| 330 | other | DTP/NCI | Cell | NCI In Vivo Anticancer Drug Screen. Data for tumor model P388 Leukemia (intraperitoneal) in CD2F1 (CDF1) mice |
| 125 | confirmatory | DTP/NCI | Cell | NCI human tumor cell line growth inhibition assay. Data for the HL-60(TB) Leukemia cell line |
| 540 | confirmatory | NCGC | Cell | Cell Viability - N2a |
| 81 | confirmatory | DTP/NCI | Cell | NCI human tumor cell line growth inhibition assay. Data for the SW-620 Colon cell line |
| 79 | confirmatory | DTP/NCI | Cell | NCI human tumor cell line growth inhibition assay. Data for the HCT-116 Colon cell line |
| 121 | confirmatory | DTP/NCI | Cell | NCI human tumor cell line growth inhibition assay. Data for the K-562 Leukemia cell line |
| 41 | confirmatory | DTP/NCI | Cell | NCI human tumor cell line growth inhibition assay. Data for the PC-3 Prostate cell line |
| 107 | confirmatory | DTP/NCI | Cell | NCI human tumor cell line growth inhibition assay. Data for the OVCAR-5 Ovarian cell line |
| 43 | confirmatory | DTP/NCI | Cell | NCI human tumor cell line growth inhibition assay. Data for the DU-145 Prostate cell line |
| 27 | confirmatory | DTP/NCI | Cell | NCI human tumor cell line growth inhibition assay. Data for the M19-MEL Melanoma cell line |
| 59 | confirmatory | DTP/NCI | Cell | NCI human tumor cell line growth inhibition assay. Data for the U251 Central Nervous System cell line |
| 85 | confirmatory | DTP/NCI | Cell | NCI human tumor cell line growth inhibition assay. Data for the MDA-MB-435 Breast cell line |

* AOM: for Activity Outcome Method

DTP/NCI: Developmental Therapeutics Program/ National Cancer Institute
NCGC: NIH Chemical Genomics Center
