# Supplementary material for: Profiling Animal Toxicants by Automatically Mining Public Bioassay Data: A Big Data Approach for Computational Toxicology
Source: PLoS One. 2014 Jun 20;9(6):e99863. doi: 10.1371/journal.pone.0099863 (PMC4064997; doi:10.1371/journal.pone.0099863)
Supplement: Table S3 — The top 32 new PubChem compounds ranked by S score. The compounds were prioritized using assay profiles which consists of 47 bioassays. Their toxicity excerpts were also listed. (DOCX) [file pone.0099863.s003.docx]

**Table S3**. The top 32 new PubChem compounds ranked by S score. The compounds were prioritized using assay profiles which consists of 47 bioassays. Their toxicity excerpts were also listed.

|  | Structure | S Score | Compound Name | Compound Type | Toxicity Excerpts | Resource |
| --- | --- | --- | --- | --- | --- | --- |
| 1 |  | 44 | Vinblastine Sulfate | Antimicrotubule [drug](http://en.wikipedia.org/wiki/Medication) to treat certain kinds of [cancer](http://en.wikipedia.org/wiki/Cancer) | Intrathecal administration of **vinblastine** sulfate injection usually results in death. | HSDB |
| 2 |  | 44 | Colchicine | Drug to treat gout | Gastrointestinal symptoms, fibrinolytic activity and injury top the liver and bone marrow | HSDB |
| 3 |  | 44 | Dactinomycin | Traditional chemotherapy drug | Overdosage of **dactinomycin** produces nausea, vomiting, diarrhea, mucositis including stomatitis, GI ulceration, skin disorders, exanthema, desquamation and epidermolysis, severe hematopoietic depression, veno-occlusive disease, and acute renal failure | HSDB |
| 4 |  | 44 | Camptothecin Sodium | Natural product | [cytotoxic](http://en.wikipedia.org/wiki/Cytotoxic) [quinoline](http://en.wikipedia.org/wiki/Quinoline) [alkaloid](http://en.wikipedia.org/wiki/Alkaloid) which [inhibits](http://en.wikipedia.org/wiki/Enzyme_inhibitor) the [DNA](http://en.wikipedia.org/wiki/DNA) [enzyme](http://en.wikipedia.org/wiki/Enzyme) [topoisomerase I](http://en.wikipedia.org/wiki/Topoisomerase_I) (topo I) | Current medicinal chemistry **14** (19): 2024–32 |
| 5 |  | 43 | Chromomycin A3 | Glycosidic antibiotic | Related to Neurodegenerative Diseases | CTD |
| 6 |  | 43 | Idarubicin Hcl | Anthracycline antibiotic | May cause myocardial toxicity | Medical Dictionary |
| 7 |  | 43 | 10-Hydroxycamptothecin | Camptothecin derivative | A topoisomerase inhibitor for cancer therapy | Chemicalbook.com |
| 8 |  | 43 | 1-Arabinofuranosyl-5-Fluorouracil | Cancer chemotherapeutic agents |  | TOXLINE |
| 9 |  | 43 | Elsamicin | Anti-tumour antibiotics | Binding to DNA and cause DNA damage | TOXLINE |
| 10 |  | 43 | Dolastatin 10 | Natural cytotoxic peptide | Microtubule-inhibitory and apoptotic effects.  Grade 3 and 4 neutropenia and grade 3 neuropathy | Clin Cancer Res Nov. 2000, 6; 4205 |
| 11 |  | 43 | Bouvardin | Natural cyclic hexapeptide | Protein synthesis inhibitor tested using CHO Chinese hamster cells in vitro | Cancer Res. Dec. 1978, 38; 4415 |
| 12 |  | 43 | Rhizoxin | [Antimitotic](http://en.wikipedia.org/wiki/Mitosis) agent with anti-[tumor](http://en.wikipedia.org/wiki/Tumor) activity | Prevents formation of the [mitotic](http://en.wikipedia.org/wiki/Mitosis) [spindle](http://en.wikipedia.org/wiki/Spindle_apparatus) inhibiting cell division | Cancer Res. **46** (1): 381–385.  Anticancer Res. **19** (5B): 3985–3988. |
| 13 |  | 43 | Echinomycin | [Peptide](http://en.wikipedia.org/wiki/Peptide) [antibiotic](http://en.wikipedia.org/wiki/Antibiotic) | Related to [Acute Kidney Injury](http://toxnet.nlm.nih.gov/cgi-bin/sis/transfer?ctd+disease+D058186), [Alzheimer Disease](http://toxnet.nlm.nih.gov/cgi-bin/sis/transfer?ctd+disease+D000544) etc. | CTD |
| 14 |  | 43 | Bruceantin | A triterpene quassinoid antineoplastic antibiotic | Decreased protein and DNA synthesis.  Related to [Lymphoma](http://toxnet.nlm.nih.gov/cgi-bin/sis/transfer?ctd+disease+D008223) | NCI Drug Dictionary  CTD |
| 15 |  | 43 | Didemnin | Cyclic [depsipeptide](http://en.wikipedia.org/wiki/Depsipeptide) | Exhibited high [toxicity](http://en.wikipedia.org/wiki/Toxicity) through a high incidence of [anaphylactic reactions](http://en.wikipedia.org/wiki/Anaphylaxis) in phase II human clinical trials | J. Am. Chem. Soc. **1981**, 103, 1857-1859.  Anti-Cancer Drugs **2000**, 11, 793. |
| 16 |  | 43 | Chrysomycin A | Antibacterial, antifungal and antiviral compound | Potent inhibitors of the catalytic activity of topoisomerase II | TOXLINE |
| 17 |  | 43 | Cyanomorpholinoadriamycin | [Antineoplastic agent](http://www.ebi.ac.uk/chebi/chebiOntology.do;jsessionid=C9F57C924A65D65F772269E8B08D9CE6?chebiId=35610) | Tested positive in chinese hamster v-79 and UDS rat hepatocytes | CCRIS |
| 18 |  | 43 | 6 Alpha-Senecioyloxychaparrinone | Natural product. Anti-leukemic quassinoid | Has high growth inhibitory and insecticidal activity | Experientia, 41(3), 379-82 (English) 1985 |
| 19 |  | 43 | Daunorubicin | [Chemotherapeutic](http://en.wikipedia.org/wiki/Chemotherapeutic) drug | Toxic manifestations of **daunorubicin** incl bone marrow depression, stomatitis, alopecia, gastrointestinal disturbances, and dermatological manifestations | HSDB |
| 20 |  | 43 | Aclacinomycin A | [Anthracycline](http://en.wikipedia.org/wiki/Anthracycline) drug | Related to [Acute Coronary Syndrome](http://toxnet.nlm.nih.gov/cgi-bin/sis/transfer?ctd+disease+D054058), [Adenocarcinoma](http://toxnet.nlm.nih.gov/cgi-bin/sis/transfer?ctd+disease+D000230), etc. | CTD |
| 21 |  | 43 | N,N-Dimethyldaunomycin Hydrochloride | Analog of CID 3085106 | Tested for **cardiotoxicity in** somatic cells | JMED CHEM 22:912-918,1979 |
| 22 |  | 42 | Cinerubin A Hydrochloride | Hydrochloride of **20** | As **20** |  |
| 23 |  | 42 | Verrucarin A 9,10-Epoxide | Fungal metabolite | Show high in vivo activity against P388 mouse leukemia | Cancer Treat Rep. 1978, Oct; 62(**10**):1585-6 |
| 24 |  | 42 | Pyrazoloacridine | Synthesized anticancer agent | Cytotoxicity in noncycling cells. | Investigational New Drugs Feb 1999,17(1):43-48 |
| 25 |  | 42 | 9-Aminocamptothecin | Anticancer agent | Predictable myelosuppression was the major dose-limiting toxicity | [Ann N Y Acad Sci.](http://www.ncbi.nlm.nih.gov/pubmed/11193898) 2000;922:224-36. |
| 26 |  | 42 | Taxol | [Mitotic inhibitor](http://en.wikipedia.org/wiki/Mitotic_inhibitor) used in [cancer](http://en.wikipedia.org/wiki/Cancer) [chemotherapy](http://en.wikipedia.org/wiki/Chemotherapy) | May be associated with acute ethanol toxicity, also related to myelosuppression, peripheral or sensory neurotoxicity, and mucositis | Pharmaceutical Research **23** (6): 1243–50.  HSDB |
| 27 |  | 42 | Emetine | Drug used as both an [anti-protozoal](http://en.wikipedia.org/wiki/Anti-protozoal) and to induce [vomiting](http://en.wikipedia.org/wiki/Vomiting) | Direct toxic effect on cardiac muscle  In ... animals ... large doses produce acute lesions in heart, liver, kidney, intestinal tract, & skeletal muscles. | Lea and Febiger, New York. pp. 438–442.  HSDB |
| 28 |  | 42 | Maytansine | [Cytotoxic agent](http://en.wikipedia.org/wiki/Cytotoxic_agent) | Exhibits cytotoxicity against many tumor cell lines and may inhibit tumor growth in vivo. | NCI Drug Dictionary |
| 29 |  | 42 | 4-Demethoxydoxorubicin | Anticancer agent | Positive in genotoxicity in vitro on UDS rat hepatocytes.  Positive in cytotoxicity in vitro on Ames salmonella typhimurium cell. | CCRIS |
| 30 |  | 42 | [10,11-Methylenedioxy-20(RS)-Camptothecin](http://www.ncbi.nlm.nih.gov/sites/entrez?cmd=search&db=pcsubstance&term=%2210%2c11%2dMethylenedioxy%2d20%28RS%29%2dcamptothecin%22%5bCompleteSynonym%5d%20366358%5bstandardizedcid%5d) | Analogy of **4** | **Tested mouse epidermis in vivo** | Int J Cancer 1996 May 16;66(4):496-505 |
| 31 |  | 42 | Nogalamycin | [Anthracycline](http://en.wikipedia.org/wiki/Anthracycline) antibiotic | Highly cardiotoxic.  cytotoxic in vitro tested against Chinese hamster ovary (CHO), mouse leukemia (L1210), and mouse melanoma (B16) cells. | Cancer Res. 1981, Jan; 41(1):18-24. |
| 32 |  | 42 | Nogalomycin | Analog of **31** | As **31** | Cancer Res. 1981, Jan; 41(1):18-24. |
